# Sleep in the time of COVID-19: findings from 17000 school-aged children and adolescents in the UK during the first national lockdown

**DOI:** 10.1093/sleepadvances/zpab021

**Published:** 2022-01-19

**Authors:** Gaby Illingworth, Karen L Mansfield, Colin A Espie, Mina Fazel, Felicity Waite

**Affiliations:** 1 Sleep and Circadian Neuroscience Institute, Nuffield Department of Clinical Neurosciences, University of Oxford, Oxford, UK; 2 Department of Psychiatry, University of Oxford, Warneford Hospital, Oxford, UK; 3 Oxford Health NHS Foundation Trust, Oxford, UK

**Keywords:** adolescents, children, COVID-19, lockdown, sleep quality, sleep duration, sleep timing, wellbeing

## Abstract

**Study Objectives:**

Sleep is essential to young people’s wellbeing, yet may be constricted by the adolescent delayed sleep phase coupled with school start times. COVID-19 restrictions caused major disruptions to everyday routines, including partial school closures. We set out to understand changes in students’ self-reported sleep quality, and associations with mental wellbeing and interpersonal functioning, during these restrictions.

**Methods:**

The OxWell school survey—a cross-sectional online survey—collected data from 18 642 children and adolescents (aged 8–19 years, 60% female, school year 4–13) from 230 schools in southern England, in June–July 2020. Participants completed self-report measures of the impact of COVID-19 restrictions on sleep quality, happiness, and social relationships. Sleep timing was compared with data collected from 4222 young people in 2019.

**Results:**

Females and older adolescents were more likely to report deteriorations in sleep during the national lockdown. Regression analysis revealed that changes in happiness (β = .34) and how well students were getting on with others in their household (β = .07) predicted change in sleep quality. Students’ bedtimes and wake times were later, and sleep duration was longer in 2020 compared to the 2019 survey. Secondary school students reported the greatest differences, especially later wake times.

**Conclusions:**

During COVID-19 restrictions, sleep patterns consistent with adolescent delayed sleep phase were observed, with longer sleep times for secondary school students in particular. Perceived deteriorations in sleep quality were associated with reductions in happiness and interpersonal functioning, highlighting the importance of including sleep measures in adolescent wellbeing research.

**Statement of Significance**

Sufficient duration and quality of sleep are essential to young people’s wellbeing. This study investigated self-perceived changes in sleep during the COVID-19 pandemic, including partial school closure. During these adapted learning conditions, when most students did not need to travel, bed and wake times were later, and sleep duration extended, compared to a pre-pandemic survey. The implication, consistent with previous research, is that delaying the start of the school day may have a beneficial impact on sleep. Disrupted sleep was associated with reductions in happiness and interpersonal functioning. This study cannot confirm direction of effect, but sleep measures should be included in adolescent wellbeing research.

## Introduction

In response to the COVID-19 pandemic, government restrictions were first imposed in the UK from March 2020. This included closing schools, except for the children of essential workers or those classed as “vulnerable”. These restrictions had the potential to impact young people’s wellbeing including their sleep and mental health. A range of adverse health outcomes during the periods of school lockdown, including reduced physical activity and increased depression, have been noted in children.[1] However, sleep duration has been observed to improve from pre-schoolers through to adolescents.[2–5] We set out to determine the self-perceived changes in sleep quality and associations with mental wellbeing and interpersonal functioning in a large survey in England during these restrictions.

### The importance of sleep for mental health

Sleep is essential to the wellbeing and healthy development of young people.[6] The duration, quality, and timing of sleep are all important components of good sleep.[7–11] Disturbed and insufficient sleep are associated with compromised emotional regulation and mood,[12–14] anxiety and depression,[15,16] and risk behaviors such as substance use, self-harm, and suicidal ideation and behavior.[17,18] Adolescence is a period of biological and social transition,[19] heightened emotionality,[20] and increased susceptibility to the onset of social-emotional/mental health disorders.[21,22] While evidence suggests a complex and bidirectional relationship between sleep and mental health,[23–25] there are indications that treating sleep problems may lead to knock-on benefits in mental health outcomes.[26,27]

### Sleep in childhood and adolescence

Developmental changes are known to occur in sleep from childhood to adolescence, with a shift towards later bedtimes and sleep onset as well as a reduction in sleep duration.[6,28] Biological, psychosocial, and societal factors contribute to pediatric sleep and its disruption.[29,30] Psychosocial factors, such as use of devices with screens[31–33] and increased autonomy[34,35] can exacerbate the biological tendency for delayed sleep in adolescence[36–38] and increase the likelihood that insufficient sleep is obtained.

### The school context

When considering societal pressures, early school start times have been identified as an impediment to sleep.[39] The typical start time of lessons at primary and secondary schools in the UK is 9:00 am. Evidence suggests that delayed school start times are associated with increased sleep duration.[40–43] However, research into sleep outcomes arising from later school start times in the UK has faced challenges; for example, a randomized controlled trial could not recruit sufficient schools that would agree to delay start times.[44] Restrictions due to the COVID-19 pandemic, however, resulted in a change in the schedules of many students. This meant many did not have to wake as early to get to their lessons, usually because of reduced need to allocate time to travel to school. This provided the setting for a natural experiment into sleep patterns as students were more likely to be able to wake later and thus closer to their “preferred” or optimum wake time.

Schools play a key part in the social world of young people.[45] The shift to remote schooling for most students had the potential to disrupt social networks and contribute to isolation from peers and fuel loneliness. In contrast, home confinement may have increased contact with families/caregivers and worries about health of vulnerable family members, which in turn may have been stressful, especially for those experiencing family adversity before lockdown. However, the possibility of positive outcomes arising from lockdown-induced changes should not be ignored with suggestions that some children and adolescents experienced reduced daily stress, fewer symptoms of anxiety and depression, and improved wellbeing.[46]

We sought to investigate young people’s self-reported sleep quality during COVID-19 lockdown restrictions, specifically partial school closure, and associations with perceived changes to their mental wellbeing and interpersonal functioning. We hypothesized there would be two distinct patterns of change in sleep quality: firstly, we predicted that without the usual additional travel this would be akin to a delay to the school start time, and sleep quality would improve for many students, particularly older secondary school students (Year 10–13) who typically have a delayed sleep phase; secondly, we predicted that given the disruption and uncertainty of the COVID-19 restrictions, sleep quality would deteriorate for those reporting reduced happiness, worsening social relationships, and increased loneliness. The secondary aim was to compare self-reported sleep patterns in 2020 with comparable data from 2019. We hypothesized that, in the context of the 2020 restrictions, bedtimes and wake times would be later and sleep duration would be longer, with greater differences for older secondary school students than for primary school students and younger secondary school students.

## Methods

### Design and participants

The present study is based on self-report data taken from the OxWell School Survey [47]. This online survey collects data in England on school students’ health and wellbeing and provides summaries of results to schools and local education authorities (LEA; an organization responsible for education in a particular area). All state-maintained (non fee-paying ) and independent (fee-paying) schools (excluding special schools) in participating LEA were eligible and invited via their LEA to sign up to be involved. Just one LEA took part in 2019 and eleven LEA in southern England took part in 2020. Participating schools inform parents, with instructions on how to opt-out, and provide remaining students with login instructions. Primary school students from Year 4–6 (typical age: 8–11 years) and secondary school/college students from Year 7–13 (typical age: 11–18 years) were invited to participate during June–July 2020. In 2019, students from Year 4–6, and Year 8, 10, and 12 from schools in one county were invited to participate between May–July. The 2020 survey included questions to help understand students’ own perceptions of the impact of the COVID-19 pandemic on their wellbeing and was completed while school was in-session but during restrictions in the partial school closure period in June–July 2020.[47] Most students were receiving only remote schooling at home, but students whose parents were key workers, considered vulnerable, or approaching national examinations were offered part- or full-time places in school. The study was approved by the University of Oxford Medical Sciences Division Research Ethics Committee (Ref: R62366/RE0010).

### Procedure

The research team provided schools with information and opt-out instructions for parents, an information video for students, and login instructions with survey links.[47] Students gave online active consent/assent in order to participate. Students could choose not to answer questions without compromising their participation and the survey took approximately 30 minutes to complete.

### Measures

#### Effect of COVID-19 restrictions on sleep quality, happiness, social relationships, and loneliness.

Six items assessed students’ perceived impact of COVID-19 restrictions, termed “lockdown”, on their mental health. Each item was rated using a sliding scale that included five category labels; all questions were prefixed with “During lockdown”. To assess sleep quality students were asked “Has your sleep been worse, the same, or better than before?”, response options ranged from “much worse” to “much better”. To assess happiness, students were asked “How happy have you been feeling in general (your mental well-being)?”, response options ranged from “much worse” to “much better”. To assess social relationships, students were asked “Have you got along less well, the same or better with other people in your household?” and “Have you got along less well, the same or better with your friends?” Response options ranged from “much less” to “much better”. To assess loneliness, students were asked “Have you felt less, the same or more left out than before?” from “much less left out” to “much more left out”, and “Have you felt less, the same or more lonely than before?” from “much less lonely” to “much more lonely”. This provided continuous data for all variables (0–100) with higher scores indicating an improvement during lockdown except for self-reports of feeling left out and lonely where lower scores indicated an improvement. In addition, for all variables three discrete categories were created (e.g., worse/same/better).

### Sleep patterns

Students reported the previous night’s bedtime (to the nearest hour: between “6 pm” and “2 am or later”) and wake time that morning (to the nearest hour: between “5 am” and “10 am or later”). For sleep onset latency (SOL), students reported how long it usually took them to get to sleep by clicking on a sliding scale to provide a continuous variable (anchored at “within a few minutes” and then at hourly intervals to “4 or more hours”). In 2019, this item asked about SOL “last night”. Time in bed was calculated from the difference between bedtime and wake time. Sleep duration was estimated as the difference between sleep onset (bedtime plus SOL) and wake time. A total of 393 cases in 2020 and 94 cases in 2019 with calculated sleep durations (<3 hours) considered potentially implausible were excluded (e.g., sleep duration <0 hours). Students rated how often they had been so worried about something that they could not sleep at night using a sliding scale with category labels from “never” to “every night”. This provided continuous data (0–100) with higher scores indicating more frequent worry.

### Statistical analysis

Initial data cleaning was based on methods developed and used since 2006 by the survey provider. Participant responses were flagged for review if the completion time was shorter than 10 minutes. Participants were excluded from the sample if: they spent under 4 minutes answering questions, answered fewer than 50% of the essential non-contingent questions in the first sections (demographics, wellbeing), or gave unrealistic responses that suggested no engagement with the survey. Descriptive statistics are presented as frequencies and percentages for categorical variables, and means and standard deviations for continuous variables. Participants in 2020 were grouped into three-year group categories for analyses (primary school students (Year 4–6); younger secondary school students (Year 7–9); older secondary school students (Year 10–13)).

Chi-square tests were performed to investigate associations between categorical variables. These examined associations between (self-perceived) change in sleep quality (three categories: worse, same, better) with student characteristics (gender, year group, whether attended school in person) and with change in happiness, social relationships, and loneliness during COVID-19 restrictions (each with three categories). A hierarchical multiple regression with list-wise deletion was conducted to investigate the relationship between change in sleep quality (continuous outcome variable) with gender, year group (categorical predictors), and the other lockdown change variables (continuous predictors described above). Gender and year group were entered at step one while lockdown change variables were entered simultaneously at step two.

To assess whether sleep patterns (sleep timing and sleep troubled by worrying) differed significantly from 2019 to 2020, independent *t*-tests with pairwise deletion were conducted. Participants were categorized into three groups to enable comparison between year groups that were included in both surveys (primary school students (Year 4–6); younger secondary school students (Year 8); older secondary school students (Year 10 & 12)). It was not possible to link participants for comparison across the two years. To test whether the patterns of effects in the results of the *t*-tests reflected the fact that different schools took part in the 2019 and 2020 surveys, we also conducted posthoc sensitivity analyses including only schools and year groups that took part in both survey years (8 schools in one county: Oxfordshire). We ran two univariate analyses of variance on the outcomes “Time in bed” and “SOL”, with the fixed categorical factors “Survey Year”, “Year Group”, and “School”, in models testing all main effects, and the interaction between “Survey Year” and “Year Group”.

Statistical analyses were performed with SPSS 27 (IBM, USA). Due to the large sample size included in the study and number of tests conducted, a *p*-value <.001 was interpreted as indicating statistical significance.

## Results

### Sample characteristics

A total of 22 336 logins were recorded in 2020, from which very short logins by students or test logins by teachers (*n* = 2130) and other partial responses indicating lack of engagement with the survey (*n* = 1167) were removed, and a further 397 logins from students attending further education colleges were not included in this analysis. There were 4369 logins in 2019, of which 127 were < 4 minutes and a further 20 rejected due to lack of engagement with the survey. Data are included from 18 642 participants (60% female, aged 8–19 years) from 230 schools in 2020 and 4222 participants (54% female, aged 8–18 years) from 36 schools in 2019. Table 1 shows sample characteristics for 2020 and Table 2 presents characteristics for 2019. The majority (90%) reported at the time of being surveyed in 2020, they had not left their home to attend in-person school regularly during the COVID-19 restrictions and partial school closures.

**Table 1.** Student characteristics for sample in 2020

Characteristic	n	% of total sample (18,642)	% who ever experienced food poverty	Mean age in years (*SD*)
Year group				
Year 4–6:				
Year 4	974	5.2	17.2	
Year 5	1151	6.2	14.4	
Year 6	1631	8.7	18.1	
Total	3756	20.1		
Age	3724			10.0 (0.88)
Year 7–9:				
Year 7	3518	18.9	10.5	
Year 8	3204	17.2	8.5	
Year 9	2905	15.6	9.2	
Total	9627	51.6		
Age	9592			12.8 (0.90)
Year 10–13:				
Year 10	2696	14.5	10.4	
Year 11	942	5.1	10.2	
Year 12	1365	7.3	8.6	
Year 13	256	1.4	5.9	
Total	5259	28.2		
Age	5248			15.7 (1.05)
Gender				
Female	11 059	59.7	10.7	
Male	7471	40.3	11.4	
Attend school during lockdown				
No: not at all/once or twice/sometimes	15 655	90.4		
Yes: most days/every day	1666	9.6		

Participants are grouped into year group categories: primary (Year 4–6); early secondary/younger adolescents (Year 7–9); late secondary/older adolescents (Year 10–13). As a measure of socio-economic deprivation, percentages are given of those who indicated that they had ever gone to bed or school hungry because there had not been enough food in the house.

**Table 2.** Student characteristics for sample in 2019

Characteristic	n	% of total sample (4222)	% who ever experienced food poverty	Mean age in years (*SD*)
Year group				
Year 4–6:				
Year 4	744	17.6	33.5	
Year 5	803	19.0	34.4	
Year 6	844	20.0	28.7	
Total	2391	56.6		
Age	2355			9.9 (0.9)
Year 8	878	21.2	18.2	
Age	872			12.8 (0.4)
Year 10 & 12:				
Year 10	624	14.8	20.4	
Year 12	329	7.8	15.4	
Total	953	22.6		
Age	953			15.5 (1.1)
Gender				
Female	2287	54.4	24.1	
Male	1917	45.6	28.7	

Participants are grouped into year group categories for comparison with the 2020 data: primary (Year 4–6); early secondary/younger adolescents (Year 7–9); late secondary/older adolescents (Year 10–13). As a measure of socio-economic deprivation, percentages are given of those who indicated that they had ever gone to bed or school hungry because there had not been enough food in the house.

### The self-perceived impact of COVID-19 restrictions on sleep quality, happiness, social relationships, and loneliness

Chi-square tests assessing associations between self-reported changes in sleep quality and other self-reported changes during COVID-19 restrictions are presented in Table 3. Self-perceived change in sleep quality was significantly associated with gender, with a pattern of deteriorated sleep for females and improved sleep for males: 44% of females experienced worse sleep compared with 31% of males, while 28% of females and 40% of males experienced better sleep (*p* < .001). Change in sleep quality was significantly associated with year group so that older year groups (Year 10–13) were more likely to have worse sleep (47%) compared with younger year groups (Year 4–6: 32%; Year 7–9: 37%) (*p* < .001). Students reported a similar change in sleep quality regardless of whether they attended school in person regularly (worse: 40%; better: 30%) or not (worse: 39%; better: 33%) (*p* = .011). Change in sleep quality was significantly associated with students’ ratings of change in happiness, social relationships, and loneliness during lockdown (*p* < .001). Students who reported they were less happy in general were more likely to perceive their sleep quality as worse (62%), while those who were happier were more likely to perceive their sleep quality as better (49%). When considering students’ relationships, worse sleep quality was more commonly reported by those who were getting on less well with other people in their household (57%) and those who were getting on less well with their friends (51%). In contrast, students who were getting on better with other people in their household were more likely to report an improvement in sleep quality (42%). When students felt more left out, they were more likely to report worse sleep quality (51%) as was the case when they felt more lonely (52%).

**Table 3.** Self-reported change in sleep quality and associations with student characteristics and other self-reported changes during lockdown

Variable	Sleep quality				
	Worse	Same	Better	n	p
Gender, *n* (%)				16 811	<.001
Female	4481 (44.2)	2814 (27.7)	2854 (28.1)		
Male	2076 (31.2)	1946 (29.2)	2640 (39.6)		
Year group, *n* (%)				16 914	<.001
Year 4–6	1091 (32.5)	1149 (34.2)	1120 (33.3)		
Year 7–9	3284 (37.4)	2534 (28.9)	2961 (33.7)		
Year 10–13	2225 (46.6)	1097 (23.0)	1453 (30.4)		
Attend school regularly, *n* (%)				15 775	.011
No	5514 (38.6)	4023 (28.2)	4731 (33.2)		
Yes	599 (39.7)	463 (30.7)	445 (29.5)		
Happiness, *n* (%)				16 355	<.001
Less happy	3532 (62.1)	1063 (18.7)	1091 (19.2)		
Same	1590 (30.0)	2108 (39.8)	1597 (30.2)		
More happy	1287 (23.9)	1451 (27.0)	2636 (49.1)		
Relations with household, *n* (%)				15 653	<.001
Less well	1843 (56.9)	611 (18.9)	787 (24.3)		
Same	2532 (35.0)	2513 (34.8)	2179 (30.2)		
Better	1687 (32.5)	1348 (26.0)	2153 (41.5)		
Relations with friends, *n* (%)				15 466	<.001
Less well	1242 (50.6)	509 (20.7)	705 (28.7)		
Same	2859 (35.2)	2685 (33.1)	2579 (31.7)		
Better	1874 (38.3)	1245 (25.5)	1768 (36.2)		
Feeling left out, *n* (%)				15 188	<.001
Less left out	2152 (37.3)	1541 (26.7)	2074 (36.0)		
Same	2308 (34.9)	2273 (34.4)	2023 (30.6)		
More left out	1441 (51.2)	551 (19.6)	825 (29.3)		
Loneliness, *n* (%)				15 384	<.001
Less lonely	1474 (33.9)	1175 (27.1)	1694 (39.0)		
Same	1781 (30.9)	2157 (37.4)	1825 (31.7)		
More lonely	2753 (52.2)	1065 (20.2)	1460 (27.7)		

Chi-square tests were performed to investigate associations between categorical variables. The percentages displayed are those calculated after excluding missing values.

A hierarchical multiple regression investigated whether the perceived change variables were associated with a change in self-reported sleep quality ratings after controlling for gender and year group. Results are shown in Table 4. Gender and year group accounted for 3% of variance in the change in sleep quality ratings during lockdown in step one. Inclusion of the perceived change variables to the model in step two explained an additional 14% of variance. Self-reported change in happiness and how students were getting on with other people in their household were significant predictors of change in sleep quality during lockdown. However, examining standardized beta values, perceived change in happiness was the strongest predictor (β = .34): feeling happier in general increased the likelihood of reporting better sleep quality.

**Table 4.** Hierarchical regression analysis of gender, year group, change in happiness, social relationships and feeling left out/lonely as predictors of change in sleep quality during lockdown in 2020

	B	SE *B*	β	*R* ^*2*^	Δ*R*^*2*^	*p*
Step 1				0.03		<.001
Gender	7.91	0.46	0.14			<.001
Year group	–0.96	0.11	–0.08			<.001
Step 2				0.17	0.14	<.001
Gender	5.82	0.43	0.11			<.001
Year group	–0.26	0.10	–0.02			.01
Happiness	0.34	0.01	0.34			<.001
Relations with household	0.09	0.01	0.07			<.001
Relations with friends	0.00	0.01	0.00			.76
Feeling left out	0.00	0.01	0.00			.87
Feeling lonely	–0.02	0.01	–0.02			.02

*n* = 13 691; Gender coded 0 = female, 1 = male; β = standardized beta coefficients

*R* ^*2*^ for Step 1, Δ*R*^*2*^ for Step 2.

### Comparison of self-reported sleep patterns: 2019 versus 2020

In comparison to the 2019 survey, bedtimes reported by respondents to the 2020 survey were significantly later (*p* < .001) as were wake times (*p* < .001). In comparison to the 2019 survey, time in bed was significantly longer in 2020 (*p* < .001) as was sleep duration (*p* < .001). However, SOL only differed significantly between 2019 and 2020 for older adolescents (Year 10 & 12: *p* < .001). Differences in self-reported sleep patterns from 2019 and 2020 are presented in Table 5.

**Table 5.** Means and standard deviations for sleep patterns in 2019 and 2020

	2019 Yr 4–6 M (SD)	2020 Yr 4–6 M (SD)	t	2019 Yr 8 M (SD)	2020 Yr 8 M (SD)	t	2019 Yr 10&12 M (SD)	2020 Yr 10&12 M (SD)	t	2020 Yr 7–9 M (SD)	2020 Yr 10–13 M (SD)
Bedtime (hh:mm)	21:08 (1:15)	21:20* (1:15)	5.92	22:17 (1:18)	22:40* (1:28)	6.96	23:11 (1:14)	23:47* (1:27)	12.48	22:38 (1:30)	23:52 (1:27)
Wake time (hh:mm)	6:49 (0:49)	7:16* (1:04)	17.81	6:40 (0:45)	7:58* (1:14)	38.45	6:45 (0:50)	8:04* (1:18)	38.48	7:53 (1:14)	8:10 (1:20)
SOL (min)	67.40 (69.41)	62.78 (57.02)	2.67	63.39 (63.35)	67.76 (57.86)	1.82	56.07 (56.85)	64.17* (57.70)	3.85	67.37 (58.40)	64.73 (57.60)
Time in bed (hh:mm)	9:41 (1:26)	9:56* (1:30)	6.07	8:23 (1:25)	9:19* (1:36)	15.68	7:35 (1:23)	8:16* (1:35)	12.74	9:15 (1:36)	8:18 (1:35)
Sleep duration (hh:mm)	8:43 (1:45)	9:02* (1:41)	6.65	7:35 (1:34)	8:21* (1:46)	11.32	6:55 (1:28)	7:26* (1:41)	8.67	8:17 (1:46)	7:27 (1:40)
Worry stopping sleep	32.25 (29.02)	29.14* (26.79)	4.08	32.34 (29.05)	34.21 (26.43)	1.67	38.17 (27.76)	38.95 (26.28)	0.75	32.57 (26.39)	39.08 (26.14)

SOL: Sleep onset latency; Worry stopping sleep measure: higher scores indicate worry more frequently stops sleep (0–100).

2019 *N* (min–max): Year 4–6 (2033–2333); Year 8 (742–869); Year 10 & 12 (807–942).

2020 *N* (min–max): Year 4–6 (3238–3710); Year 8 (2746–3177); Year 10 & 12 (3527–4024); Year 7–9 (8197–9526); Year 10–13 (4579–5212).

Independent t-tests were used to assess differences for year groups (Year 4–6, Year 8, Year 10 & 12) between 2019 and 2020.

*p < .001.

**Figure 1.**
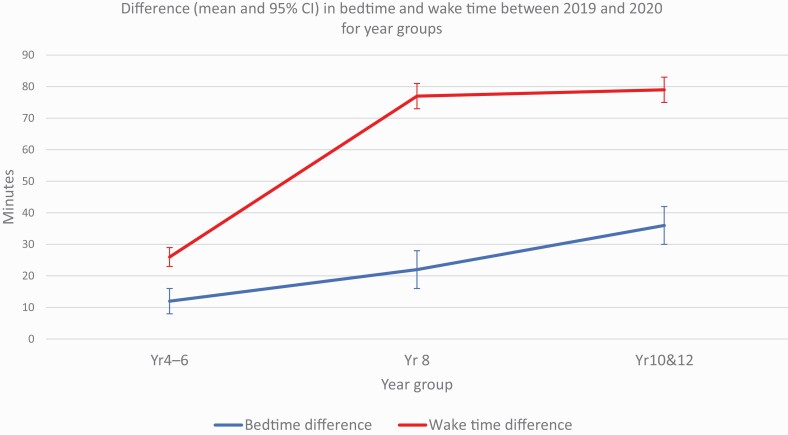
Difference (mean and 95% CI) in average bedtime and wake time between 2019 and 2020 for year groups.

**Figure 2.**
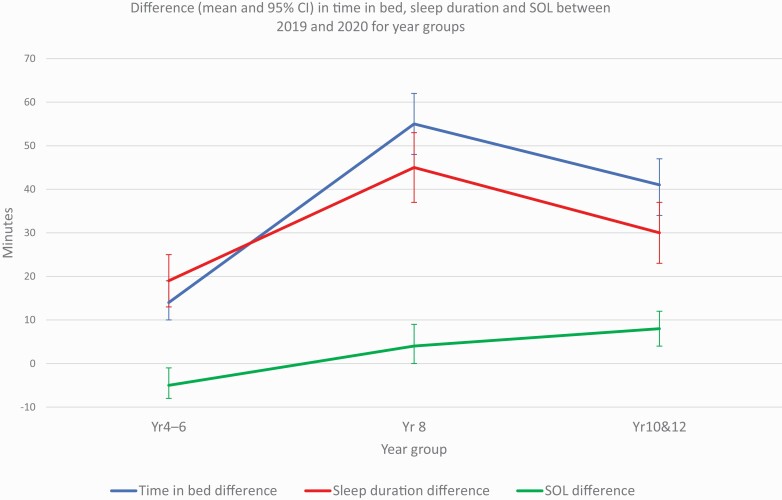
Difference (mean and 95% CI) in time in average bed, sleep duration, and SOL between 2019 and 2020 for year groups.

Figures 1 and 2 show the mean difference in sleep timing average between 2019 and 2020 by year group category. Primary school students (Year 4–6) who took part in 2020 went to bed later (12 mins; 95% CI, 8–16 mins), had later wake times (26 mins; 95% CI, 23–29 mins), longer time in bed (14 mins; 95% CI, 10–19 mins) and longer sleep durations (19 mins; 95% CI, 13–25 mins) than respondents from the same year groups in 2019. Younger secondary school students (Year 8) who took part in 2020, went to bed later (22 mins; 95% CI, 16–28 mins), had later wake times (1 hr, 17 mins; 95% CI, 1 hr 13 mins–1 hr, 21 mins), longer time in bed (55 mins; 95% CI, 48 mins–1 hr, 02 mins) and longer sleep durations (45 mins; 95% CI, 37–53 mins) than respondents who were in Year 8 in 2019. Older secondary school students (Year 10 & 12) who took part in 2020 went to bed later (36 mins; 95% CI, 30–41 mins), had later wake times (1 hr, 19 mins; 95% CI, 1 hr, 15 mins–1 hr, 23 mins), longer time in bed (41 mins; 95% CI, 34–47 mins) and longer sleep durations (30 mins; 95% CI, 23–37 mins) than respondents from the same year groups in 2019. When comparing the frequency that worrying prevented students’ sleep in 2019 and 2020, the only significant difference was found for students in Year 4–6 whereby students who took part in 2020 were less often too worried to sleep than students who took part in 2019 (–3.11; 95% CI, –4.61 to –1.62; *t*(4340.02) = –4.08, *p* < .001).

Results of sensitivity analyses including only schools involved in the survey in both 2019 and 2020 demonstrated a similar pattern to the *t*-test results comparing sleep patterns in 2019 and 2020. Please see the Supplementary Materials.

## Discussion

The aim of this study was to investigate children and adolescents’ perceived impact of COVID-19 restrictions on their sleep during partial school closures in the UK, and to compare sleeping behavior in 2020 with comparable data from 2019. We found a strong association between students’ perceived changes in the quality of their sleep and in their general happiness (mental wellbeing) over the course of the first UK lockdown. Comparing the large sample of students surveyed in 2020 with responses from schools surveyed in 2019, we saw differences in the duration and timing of sleep patterns – with greater differences for adolescents than children. For example, students in Years 10 and 12 were on average sleeping half an hour longer each night. The altered sleep pattern is consistent with studies across other countries experiencing pandemic-related restrictions and those using parental reports of sleep duration.[1–5] Together these findings underscore the importance of sleep for mental wellbeing and the potential impact of school start times on the opportunity for sleep, particularly for secondary school students.

### Sleep quality

It is well established that sleep changes from childhood to adolescence[6,28,29] and we expected to find evidence that sleep quality would be associated with year group. Findings from this study support our hypothesis that without the usual pressures to get ready for and travel to in-person school, sleep quality would improve for many students, with a third of those in primary school and early secondary school reporting that their sleep quality was better during lockdown. However, contrary to our prediction that older secondary school students might benefit most from not having to adhere to the usual morning routines and timings, it was these students (Year 10–13) who were more likely to experience worse sleep with almost half of them reporting their sleep had deteriorated. During these particular school years in the UK, students study for and take public examinations (for example, GCSEs and A-levels). One explanation is that the academic disruption and uncertainty surrounding these examinations during the pandemic may have adversely affected the sleep quality of older secondary school students. This is in line with findings reported elsewhere from this survey, that students in Year 10 and 12 (with public examinations scheduled to take place the following year) had increased odds of reporting self-perceived deterioration to their wellbeing during lockdown, as well as increased odds of depression and anxiety.[48]

This study adds to the large body of work supporting the association between sleep and mental wellbeing.[12,14,15,16,49] Our findings reveal that young people’s self-reported change in sleep quality was associated with a self-perceived change in their happiness during lockdown, and support the prediction that deteriorations in sleep quality would be associated with reduced happiness. Moreover, when all lockdown change variables were considered together in a regression analysis, after controlling for covariates (year group and gender), it was happiness that best predicted sleep quality. Overall, we propose that sleep quality must be integral to our assessment of the wellbeing of young people. Although this study was not designed to investigate the mechanisms of a prospective relationship between sleep quality and mental wellbeing, findings suggest it is important to address how well adolescents are sleeping when they experience lowered levels of happiness. Sleep problems often co-occur with anxiety and depression, and sleep quality at age 15 has been found to predict later anxiety and depression.[16] Strikingly, a recent epidemiological meta-analysis including 192 studies across the globe, found that for a third of individuals, the onset of first mental illness occurs before 14 years of age and before 18 years in almost half of all individuals.[22] Preventative approaches and early interventions for mental health should therefore take sleep into consideration.

In line with our prediction that a perceived deterioration to sleep would be associated with negative perceived changes to interpersonal functioning (social relationships and loneliness) during lockdown, approximately half of the students who perceived they were getting on less well with other people in their household or less well with their friends, also rated their sleep quality to be worse. In a related finding, approximately half of the students who reported feeling more left out or lonelier reported worse sleep quality. Conversely, the benefit of good interpersonal functioning was demonstrated by the finding that those who were getting on better with others in their household, were more likely to perceive their sleep quality had improved. Regression analysis revealed that perception of household relations was the only measure of interpersonal functioning to be significantly associated with sleep quality after controlling for covariates (year group and gender). Household environment[31,50] and family demands,[51] have been found to be associated with adolescent sleep. Intuitively, the association between sleep quality and household relations is unsurprising given how students were more confined to their homes during the period of COVID-19 lockdown restrictions.

We see a distinct pattern of gender difference, with much higher numbers of female participants reporting deteriorated sleep: nearly half of females, compared to a third of male participants, reported worsening sleep. This pattern is reversed with self-reported improvements in sleep: 40% of males reported better sleep during lockdown compared with 28% of female participants. This finding is consistent with gender differences in rates of insomnia in adults.[52] Yet it also raises questions about the differential impact of the pandemic, and associated restrictions, across genders. In an Icelandic study that investigated the impact of the pandemic on mental health, adolescent girls were found to have greater increases in depressive symptoms and decreases in mental wellbeing than boys.[53] It is likely that further mixed-methods research will be able to determine why these differences might have been observed.

### Sleep duration and timing

This study represents a natural experiment into the sleep patterns of children and adolescents in the absence of early school routines. It provides an insight into sleep when there was a greater possibility that individuals could sleep at their preferred times and possibly bringing them closer to their optimal durations. Consistent with existing research conducted during the pandemic[1–5] our findings support the hypothesis that when comparing sleep timing during lockdown in 2020 with that of the usual school schedule of the previous year, students’ bedtimes and wake times would be later, and time in bed and sleep duration would be longer. Consistent with maturational changes and delayed sleep-wake behavior,[36–38] reported time spent in bed by adolescents in secondary school was longer in 2020 compared to responses from the 2019 survey, demonstrating greater differences between survey years than seen for children in primary school. Although care should be taken when comparing between survey years – due to differences in the two population samples – a sensitivity analysis using a smaller sample with matched schools replicated the pattern found with the complete samples. Notably, wake times showed the largest difference with an average delay of 1 hour, 17 minutes for younger secondary school students and 1 hour, 19 minutes for older secondary school students compared with an average bedtime delay of 22 minutes for younger secondary school students and 36 minutes for older secondary school students. These results indicate that a short delay to bedtimes was compensated by a much longer delay to wake times. The delay in wake times is consistent with the majority of studies investigating later school start times.[40] Our findings suggest that delaying the school day in the UK may have the potential to allow young people to wake later, thereby increasing sleep duration. Indeed, organizations in the US, such as the American Academy of Pediatrics,[54] recommend that middle and high schools should begin no earlier than 8:30am.

An important finding is that sleep duration was longer during lockdown for all year groups, supporting evidence that has emerged from other countries. An increase was found in a sample of 600 adolescents (aged 10–19 years) in Palestine when 67% reported sleeping more during lockdown compared to before.[55] Furthermore, a qualitative study in Canada found that adolescents (mean age: 13.5 years) reported longer sleep durations during the school shutdown[56] as did a sample of adolescents (aged 15–17 years) reporting on school nights during COVID-19 restrictions in the US.[57] However, it is noteworthy that when comparing the average self-reported sleep duration from our survey in 2020 with the National Sleep Foundation sleep duration guidelines,[58] only primary school children reached the recommended amount for their age group (9–11 hours), while secondary school students still remained approximately 37 minutes below the minimum recommended period of time.

Despite the fact that school closures meant wake times could be later and sleep duration could increase for many adolescents, the subjective experience for some was that their sleep quality was worse. Although speculative, a reduction in sleep quality could have been due to increased stress levels – there are many clear potential contributors to increased stress including the context of the pandemic, as well as unprecedented changes to national examinations taken by 16–18-year-olds in the UK. This interpretation is also in line with the findings that students in Years 10 and 12 were more likely to have high depression and anxiety scores and to perceive deteriorations to their wellbeing during lockdown.[48] Understanding whether sleep quality improves again after the restrictions lift, or if there are persistent problems will be important for identifying those who might benefit from interventions targeting sleep. In addition, although this study asked about subjective changes to sleep, no measure of clinical sleep problems, such as insomnia, was included. Therefore, we do not know if these changes to sleep were problematic or indicative of a clinical level of disturbance.

Our findings should be interpreted within the context of certain limitations. These data stem from a cross-sectional design and so any interpretation does not allow an exploration of causality. The current study cannot accurately measure the impact of the restrictions during the pandemic on children and adolescents’ sleep or wellbeing, as linked pre-pandemic measures per participant are not available. Instead, it assesses students’ self-perceived changes to these factors in 2020, and uses an earlier survey from a comparable (but smaller) sample of students in 2019 as a pre-pandemic reference/baseline measure. It should be noted also that the analyses exclude confounding factors and therefore interpretation of the findings should be taken with caution and consideration of the wider context. Therefore, differences between sleeping patterns in 2019 and 2020 could to some extent reflect differences in the characteristics of each sample, perhaps more in some year groups than others. Both the 2019 and the 2020 surveys are prone to selection and nonresponse bias as only some LEA were engaged, only some schools in each LEA responded to the invitation to take part, some students were opted out by parents, and other students might have been unable or unwilling to complete the survey. The 2020 sample includes more LEA in Southern England but is less representative than the 2019 sample, as the response rate per year group in each school and the self-reported food poverty was lower in 2020; many students might not have had access to digital devices or have been in good contact with their schools during the partial school closures. The 2019 sample was collected in-school during lesson periods, with approximately 85% of each invited school class taking part, while the 2020 survey was more prone to nonresponse bias due to the majority survey links being sent out to students at home. In 2020, many younger students could only be contacted by school via their parents, apart from the few who were offered in-school places. Therefore, we provide a sensitivity analysis for the 2019–2020 comparisons selecting only the schools and year groups that took part in both surveys.

The measures used were not validated, which can lead to measurement bias. The measures of “perceived change” to sleep and other factors in the 2020 survey were designed rapidly in response to the onset of lockdown, but aiming to be symmetrically worded (around “same”, i.e. no change), not suggestive of either positive or negative changes during lockdown. The measures of self-reported sleep timing were inherited from earlier surveys and retained for potential comparison, with potential validity through working practice though not published as an instrument. Measuring interpersonal functioning by self-report is particularly difficult with younger students, and although these measures are not validated, and required both introspection and retrospection from young students, their development incorporated advice from school staff and healthcare providers. In addition, it has been noted that objective measures of sleep timing do not predict child ratings of sleep quality[59] and it is the subjective reports of sleep disturbance during childhood and adolescence that seem to predict wellbeing difficulties in the long term.[60,61] Future survey research of this kind may act as a pathway to explore sleep and wellbeing using validated self-report scales in subsamples to identify those who may be most in need of intervention.

Information on school schedules is not recorded, which limits our interpretation of the impact of school start times. Students were able to report their wake time and bedtime as whole hours (e.g., 7 am/10 pm) rather than in increments and this may have reduced the accuracy of sleep timing. We did not investigate social jetlag,[11] the misalignment of biological and social time, as information on sleep timing at weekends was not collected. For example, a study in India found that young people’s social jetlag reduced in lockdown.[62]

## Conclusion

Despite the longer sleep times reported in the 2020 survey compared to the 2019 survey, many secondary school students perceived that their sleep had deteriorated during COVID-19 restrictions and school closures. This pattern was most striking in older students and females. In contrast, fewer of the younger students perceived negative impacts on their sleep quality during the lockdown. Sleep quality was associated with perceived changes to happiness: highlighting the importance of accounting for sleep quality in studies investigating young people’s wellbeing.

## Supplementary Material

zpab021_suppl_Supplementary_Materials

## Data Availability

Fully de-identified extracts of the data can be provided to academic research collaborators upon reasonable request, following a review process by the research team to ensure uses of the data fall under the remit of the intended purposes set out in the privacy information and to prevent duplication of analyses. The data are not publicly available due to ethical and information governance restrictions. The full list of questions and other details are available on a project-specific “OxWell” OSF website along with publication of the study protocol (see https://osf.io/sekhr/). Full data dictionaries can be made available upon approval for access to data extracts.
