# Supplementary material for: Sleep in the time of COVID-19: findings from 17000 school-aged children and adolescents in the UK during the first national lockdown
Source: Sleep Adv. 2022 Jan 19;3(1):zpab021. doi: 10.1093/sleepadvances/zpab021 (PMC8807290; doi:10.1093/sleepadvances/zpab021)
Supplement: zpab021_suppl_Supplementary_Materials [file zpab021_suppl_supplementary_materials.docx]

**Sleep in the time of COVID-19: Findings from 18000 school-aged children and adolescents in the UK during the first national lockdown**

Gaby Illingworth^1a^, Karen L. Mansfield^2a^, Colin A. Espie^1^, Mina Fazel^2b^ and Felicity Waite^2, 3b*^

^1^ Sleep and Circadian Neuroscience Institute, Nuffield Department of Clinical Neurosciences, University of Oxford, Oxford OX1 3QU, UK

^2^Department of Psychiatry, University of Oxford, Warneford Hospital, Oxford OX3 7JX, UK

^3^ Oxford Health NHS Foundation Trust, Oxford OX3 7JX, UK

^a^ Joint first authors

^b^ Joint last authors

^*^Corresponding author. Felicity Waite, Department of Psychiatry, University of Oxford, Warneford Hospital, Oxford OX3 7JX, UK. Email: felicity.waite@psych.ox.ac.uk

**Supplementary materials**

*Matched Schools Analysis 2019:2020*

The differences between 2019 and 2020 for ‘Time in bed’ and ‘SOL’ were partially supported by sensitivity analyses that included only responses from students in the schools and year groups that took part in both 2019 and 2020 (see supplementary tables 1 and 2). In these more closely matched samples, students in the 2020 survey reported spending more time in bed (545 minutes) compared to the 2019 survey (513 minutes), *F*_1, 2418_ = 25.8, *p <* .001, *η_p_^2^* = .011. Time in bed also differed significantly between the three age groups (*F*_2, 2418_ = 89.7, *p <* .001, *η_p_^2^* = .069), but differences between the 8 schools were not significant (*F*_7, 2418_ = 1.8, *p =* .078, *η_p_^2^* = .005). Although the interaction between Survey Year and Year Group did not reach significance (*F*_2, 2418_ = 2.8, *p =* .063, *η_p_^2^* = .002), the pattern when comparing this smaller sample (*N* for 2019 = 1680; *N* for 2020 = 751) was similar to the analysis using the full samples, suggesting that only secondary school students (Years 7–13) spent more time in bed in 2020. In support of this interpretation, 95% confidence intervals for time in bed (2019 versus 2020) were overlapping for primary school students in Years 4–6 but not for secondary school students in Years 8 or 10&12 (see supplementary table 1). With matched schools, SOL did not differ significantly between 2019 and 2020 (*F*_1, 2573_ = 1.0, *p =* .309, *η_p_^2^* < .001), nor between age groups (*F*_2, 2573_ = 0.4, *p =* .658, *η_p_^2^* < .001), or schools (*F*_7, 2573_ = 1.6, *p =* .123, *η_p_^2^* = .004). The interaction between Survey Year and Year Group also did not reach significance (*F*_2, 2573_ = 2.9, *p =* .055, *η_p_^2^* < .002), although the pattern was similar to the *t*-test results using the whole 2020 sample, suggesting no difference in SOL for primary school students in Years 4–6, but longer SOL in 2020 for students in secondary school Years 8 and 10&12.

**Supplementary tables**

**Supplementary Table 1:** Time in bed for schools that participated in both 2019 and 2020

| Survey year | Year group | Estimated Mean | Std. Error | 95% CI  Lower bound | 95% CI  Upper bound |
| --- | --- | --- | --- | --- | --- |
| 2019 | Yr4–6 | 9:26 | 0:18 | 8:50 | 10:02 |
|  | Yr 8 | 8:32 | 0:07 | 8:19 | 8:46 |
|  | Yr10&12 | 7:42 | 0:07 | 7:29 | 7:55 |
| 2020 | Yr4–6 | 9:35 | 0:13 | 9:08 | 10:01 |
|  | Yr 8 | 9:20 | 0:08 | 9:05 | 9:36 |
|  | Yr10&12 | 8:20 | 0:07 | 8:05 | 8:35 |

*Note:* All times are presented as hh:mm. *N* for 2019 = 1680; *N* for 2020 = 751.
Estimated marginal means are provided from an ANOVA on matched schools with survey year, year group, and school as factors.

**Supplementary Table 2:** Sleep onset latency for schools that participated in both 2019 and 2020

| Survey year | Year group | Estimated Mean | Std. Error | 95% CI  Lower bound | 95% CI  Upper bound |
| --- | --- | --- | --- | --- | --- |
| 2019 | Yr4–6 | 75.45 | 12.14 | 51.65 | 99.25 |
|  | Yr 8 | 55.87 | 4.55 | 46.96 | 64.79 |
|  | Yr10&12 | 52.11 | 4.34 | 43.60 | 60.62 |
| 2020 | Yr4–6 | 62.56 | 8.87 | 45.18 | 79.95 |
|  | Yr 8 | 66.55 | 5.21 | 56.34 | 76.76 |
|  | Yr10&12 | 67.01 | 5.00 | 57.20 | 76.81 |

*Note:* All times are presented as minutes. *N* for 2019 = 1824; *N* for 2020 = 762.
Estimated marginal means are provided from an ANOVA on matched schools with survey year, year group, and school as factors.
